# Letter to editor on “Clinicopathological characteristics and biomarker alterations in early-onset vs. late-onset colorectal cancer: a systematic review and meta-analysis”

**DOI:** 10.1097/JS9.0000000000003657

**Published:** 2025-11-04

**Authors:** Jia Tang, Mi Yan, Zhangxue Hu

**Affiliations:** Department of Pediatrics, Daping Hospital, Army Medical University, Chongqing, China

**HIGHLIGHTS**

- The regional differences in molecular spectra indicate the influence of genetic lineage, environmental exposure, and lifestyle factors.
- The limited number of studies on stratified or biomarker specific survival data during the reporting phase limits further interpretation.
- Their therapeutic significance has not been fully explored.
- Future revisions of screening guidelines should consider combining molecular risk stratification with age-based criteria to identify high-risk populations as early as possible.

*Dear Editor,*

We read with great interest the systematic review and meta-analysis by Huang *et al* (2025) comparing clinicopathological and molecular characteristics between early-onset colorectal cancer (EOCRC, <50 years) and late-onset colorectal cancer (LOCRC, ≥50 years)^[1]^. Their work integrates data from 40 studies and over 250 000 patients, providing compelling evidence that EOCRC is a unique entity with invasive histopathology, diagnostic staging, and unique molecular features, including higher TP53 mutations and high microsatellite instability (MSI-H) status.

This research has greatly promoted people’s growing awareness of EOCRC as a global public health challenge, with its incidence rate rising by 2%–3% every year^[2–4]^. The author correctly emphasizes the necessity of tailoring screening and treatment strategies. However, although meta-analysis provides valuable insights, there are several limitations – some inherent to the included studies and others related to methodological limitations – that are worth discussing to guide future research.

First, although the high heterogeneity observed across studies (with many results having an *I*^2^ > 50%) has been partially addressed through subgroup and meta regression analysis, this highlights the challenge of generalizing research findings. The regional differences in molecular spectra (such as lower BRAF/KRAS mutations in the western EOCRC population) indicate the influence of genetic lineage, environmental exposure, and lifestyle factors^[5,6]^. However, meta-analysis did not fully adjust for confounding variables such as diet, smoking, obesity, or socioeconomic status, which may contribute to these differences. Future research should incorporate standardized environmental and lifestyle data for more detailed comparisons.

Second, although the authors reported no significant difference in 5-year overall survival rates between EOCRC and LOCRC, this overall finding may mask important subtle differences. Young patients with EOCRC have a greater loss of life, and survival outcomes may vary depending on molecular subtypes and treatment methods. The limited number of studies on stratified or biomarker specific survival data during the reporting phase (e.g., only four studies on BRAF mutation EOCRC) limits further interpretation. Prospective, molecular stratified cohorts are needed to evaluate survival rates in the context of modern treatments, including immune checkpoint inhibitors for MSI-H tumors and emerging drugs targeting the TP53 pathway^[7,8]^.

Third, although the study emphasizes the high prevalence of TP53 and MSI-H in EOCRC, their therapeutic significance has not been fully explored. Although immune checkpoint inhibitors have been approved for MSI-H metastatic colorectal cancer^[7]^, their efficacy in young EOCRC patients has not been well demonstrated, especially in adjuvant or neoadjuvant environments. Similarly, in prospective trials involving young patients, there have been few studies on TP53 targeted therapies such as MDM2 inhibitors APR-246. The author’s call for “radical and tailored treatment strategies” is timely, but specific recommendations for biomarker driven therapy are limited due to the lack of intervention studies in meta-analyses.

Fourth, although the definition of EOCRC (≤50 years) is practical, it may not fully capture biological heterogeneity. The newly emerging data suggests that there are further subdivisions within EOCRC with different molecular features (e.g., EOCRC <35 years old). Future revisions of screening guidelines should consider combining molecular risk stratification (such as KRAS, TP53, and MSI status) with age-based criteria to identify high-risk populations as early as possible.

Finally, meta-analysis mainly relies on retrospective data, which are susceptible to selection bias, uneven follow-up, and unmeasured confounding factors. Only a few studies have adequately controlled for genetic syndromes (such as Lynch syndrome) or inflammatory bowel disease, which may affect molecular and clinical features. Prospective multicenter studies with unified sequencing protocols and clinical annotations are crucial for validating these findings.

In summary, Huang *et al* provided a foundational resource that affirmed the unique properties of EOCRC. Their work emphasizes the urgency of revising screening guidelines and personalized treatment methods. However, in order to translate these insights into clinical practice, future research must address these limitations through prospective, molecular informed, and global collaborative studies.

Artificial Intelligence – the TITAN guideline^[9]^.

## Data Availability

Not applicable.

## Research registration unique identifying number (UIN)

There are no conflicts of interest.
